# Circular RNAs in renal cell carcinoma: implications for tumorigenesis, diagnosis, and therapy

**DOI:** 10.1186/s12943-020-01266-7

**Published:** 2020-10-14

**Authors:** Ying Wang, Yunjing Zhang, Ping Wang, Xianghui Fu, Weiqiang Lin

**Affiliations:** 1 Kidney Disease Center, The Fourth Affiliated Hospital, Institute of Translational Medicine, Zhejiang University School of Medicine, Jinhua, 322000 Zhejiang China; 2 Department of Urology, the First Affiliated Hospital, Zhejiang University School of Medicine, Hangzhou, 310003 Zhejiang China; 3 Division of Endocrinology and Metabolism, State Key Laboratory of Biotherapy and Cancer Center, West China Hospital, Sichuan University and Collaborative Innovation Center of Biotherapy, Chengdu, 610041 Sichuan China

**Keywords:** CircRNA, Renal cell carcinoma, Biomarker, Targeted therapy

## Abstract

Renal cell carcinoma (RCC) is the most common malignant kidney tumor and has a high incidence rate. Circular RNAs (circRNAs) are noncoding RNAs with widespread distribution and diverse cellular functions. They are highly stable and have organ- and tissue-specific expression patterns. CircRNAs have essential functions as microRNA sponges, RNA-binding protein- and transcriptional regulators, and protein translation templates. Recent reports have shown that circRNAs are abnormally expressed in RCC and act as important regulators of RCC carcinogenesis and progression. Moreover, circRNAs have emerged as potential biomarkers for RCC diagnosis and prognosis and targets for developing new treatments. However, further studies are needed to better understand the functions of circRNAs in RCC. In this review, we summarize and discuss the recent research progress on RCC-associated circRNAs, with a focus on their potential for RCC diagnosis and targeted therapy.

## Background

Renal cell carcinoma (RCC), the most common kidney neoplasm, originates in renal tubular epithelial cells and accounts for 85–90% of adult renal malignancies [1]. RCC is the sixth- and eight-most common cancer in males and females, and it is estimated that there will be 73,750 new cases of RCC in 2020 in the US [2]. RCC diagnoses have been increasing in recent years, mainly due to improvements in imaging techniques and ultrasonography [3, 4]. Currently, surgical resection is the first-line treatment for RCC. However, local recurrence or distant metastasis still occur in some patients even after radical nephrectomy. Moreover, the majority of RCCs are resistant to chemotherapy and radiotherapy once they recur or metastasize [5–7]. Although targeted therapies have significantly improved therapeutic outcomes for advanced RCC patients, their effectiveness is still limited. Cancer recurrence, metastasis, and resistance to therapy affect the recovery of patients with RCC [8–13]. Therefore, the early detection of postoperative micrometastases and recurrent lesions, and overcoming RCC drug resistance can improve patient prognosis.

RCC tumorigenesis is an extremely complex process that involves genetic mutations and the dysregulation of epigenetic pathways [14–20]. The tumor suppressor von Hippel–Lindau (VHL), one of the most frequently mutated genes in RCC, regulates the hypoxia pathway by controlling the activity of hypoxia-inducible factors (HIFs) [21]. The phosphoinositide 3-kinase (PI3K)/AKT/mammalian target of rapamycin (mTOR) pathway is constitutively activated in RCC and plays a crucial role in regulating cell growth [22]. Concurrently, epigenetic changes in RCC such as DNA methylation [23], noncoding RNAs (ncRNAs), and histone modifications [24] have been extensively studied [25]. It has been demonstrated that numerous ncRNAs are dysregulated and involved in cancer initiation and progression. Thus, the potential application of targeting ncRNAs is currently an emerging area of clinical interest [26].

The discovery of ncRNAs has added to the understanding of how RCC develops and how it may be treated. It is generally recognized that most of the mammalian genome is transcribed into ncRNAs, including microRNAs (miRNAs) and long ncRNAs (lncRNAs). miRNAs, the best-studied class of ncRNAs, are involved in the pathogenesis of different cancers, including RCC. For example, miR-210 is upregulated in RCC and it regulates VHL, HIF-1, and HIF-2, which in turn promote RCC aggressiveness via multiple mechanisms [27]. Moreover, increased levels of miR-210 are directly correlated with poor patient outcomes, suggesting that it could be a potential biomarker for RCC diagnosis and prognosis [27]. The significance of miRNAs in RCC has been comprehensively summarized in a recent excellent review [28]. Accumulating evidence also suggests the involvement and potential importance of lncRNAs in gene regulation in human diseases, including RCC [29, 30]. For instance, the upregulation of the lncRNA MALAT1 is correlated with RCC progression and a poor prognosis [30].

Circular RNAs (circRNAs), a subclass of lncRNAs, are covalent single-chain closed-loop structures lacking terminal 5′ and 3′ ends [31]. Because of this extraordinary construction, circRNAs are resistant to degradation by exonucleases and are more stable than linear RNA. Thus, many studies have focused on the potential role of circRNAs as a promising disease biomarker. Further, studies in the past 10 years have highlighted the regulatory functions of circRNAs in both physiological and pathological settings [31]. Recently, the significance of circRNAs has been demonstrated in several cancers, including urinary system tumors such as bladder carcinoma [32] and prostate cancer [33]. Increasing evidence has witnessed that circRNAs play critical roles in RCC cell proliferation, apoptosis, migration, and invasion [34–50]. In this review, we summarize the circRNAs involved in RCC and their relevance to current clinical practice. A comprehensive understanding of circRNAs may provide valuable clues and useful information for future treatment options for RCC.

### Overview of circRNAs

CircRNAs were originally dismissed as an artifact of transcription [51, 52]. Starting in 2010, assisted by high-throughput sequencing technology and bioinformatics, the number and types of identified circRNAs increased rapidly. In human tissues alone, in silico studies have predicted the existence of over 30,000 circRNAs, and subsequent studies on their biogenesis, characteristics, and functional mechanisms have been carried out [53–59].

**Fig. 1:**
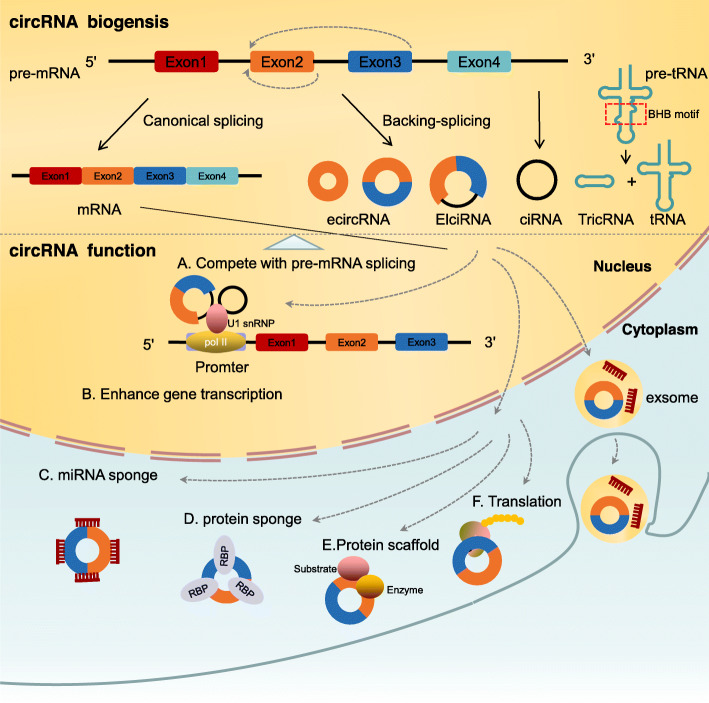
Biogenesis and functions of circular RNAs (circRNAs). The biogenesis of circRNAs involves the noncanonical back-splicing process, which generates three types of circRNAs: ecircRNAs, ciRNAs, and EIciRNAs. TricRNAs are another type of circRNA that are generated via the splicing of pre-tRNA. CircRNA biogenesis competes with linear pre-mRNA splicing. CircRNAs enhance the transcription and splicing of their parental genes by interacting with RNA pol II or U1 small nuclear snRNP. CircRNAs act as miRNA sponges to regulate the expression of relevant target genes. CircRNAs bind to RBPs to mediate their actions. CircRNAs act as protein scaffolds to promote the binding of a substrate to an enzyme. CircRNAs can be translated into proteins

In contrast to the canonical splicing of mRNAs, circRNAs are the result of a unique back-splicing process between the 5′ splice donor site and 3′ splice acceptor site, which forms a continuous loop structure (Fig. 1). Different splicing processes result in three main types of circRNAs, including exonic circRNAs (ecircRNAs), exon-intron circRNAs (EIciRNAs), and circular intronic RNAs (ciRNAs). In addition, tRNA intronic circRNAs (tricRNAs) are generated by pre-tRNA splicing [60]. circRNA biogenesis can compete with linear pre-mRNA splicing, thereby affecting the alternative splicing of linear mRNAs and leading to altered gene expression. However, some studies have also shown that EIciRNAs and ciRNAs can enhance the transcription and splicing of their parental genes by interacting with RNA polymerase II (pol II) or the essential splicing factor U1 small nuclear ribonucleoprotein (snRNP) [61, 62]. Hundreds of circRNAs, encoded by the mitochondrial genome (mecciRNA) in both human and mouse cells, were recently identified [63]. The study found that some mecciRNAs can act as molecular chaperones to facilitate mitochondrial transport of proteins encoded by nuclear genes, which is essential for mitochondrial adaptation to physiological conditions. A study by Su et al. revealed that the novel mecciRNA SCAR has a critical role in nonalcoholic steatohepatitis (NASH) pathogenesis [64]. They showed that the inter-organelle metabolic axis could be disrupted with mitochondria-targeting nanoparticles (mito-NPs), which contained circRNA-expressing vectors and triphenylphosphonium (TPP)-decorated amphiphilic cationic peptides (TACP). mito-NPs can deliver circRNA SCAR specifically to the mitochondria, thereby alleviating metaflammation by reducing mROS output. This study highlighted a novel therapeutic strategy for immunometabolic and mitochondrial-related diseases.

The potential functions and biological activities of circRNAs have been extensively studied **(**Fig. 1**)**. The best-studied function of circRNAs is to act as miRNAs sponges, thereby regulating downstream miRNA targets. For instance, circMTO1 targets miR-9, thereby inhibiting hepatocellular carcinoma proliferation and invasion by increasing p21, the target of oncogenic miR-9 [65]. Additionally, circRNAs can bind to numerous RNA-binding proteins (RBPs), acting as protein sponges or decoys to regulate protein functions [66]. A few circRNAs have been shown to serve as protein scaffolds, thereby promoting substrate-enzyme binding and affecting the kinetics of the reaction [67, 68]. Furthermore, under specific conditions, a subset of circRNAs can be translated into proteins [69], in a process that can be enhanced by the presence of N^6^-methyladenosine (m^6^A) [70], thus adding another layer of complexity to circRNA regulation.

Because of their diverse functions, circRNAs not only regulate fundamental biological processes, but also play critical roles in a wide spectrum of human diseases including cancer [71–76]. Importantly, circRNAs are resistant to exoribonucleases and are therefore stable in the circulation, which makes them promising biomarkers for cancer diagnosis and prognosis.

### circRNA profiles in RCC

The existence of circRNAs was first reported in viroids in 1976 [77]. In the past few years, research interest in circRNAs has significantly increased and many bioinformatics tools have been developed to accelerate these investigations [78–82]. Particularly, a few genome-wide transcriptional profiles of circRNAs in RCC have been reported. For instance, Ma et al. found that 542 circRNAs were aberrantly expressed in clear cell RCC (ccRCC) by using a circRNA microarray. Among these, 324 circRNAs were significantly downregulated, whereas 218 were upregulated in ccRCC tumors [83]. Using the Arraystar microarray to profile seven matched ccRCC samples, Franz et al. detected a total of 13,261 circRNAs, of which 78 circRNAs were upregulated and 91 were downregulated more than two-fold [84]. These high-throughput results strongly suggest a potential involvement of circRNAs in the pathogenesis of RCC, although their functions and underlying mechanisms are still being elucidated. Of note, different deregulated circRNAs were characterized in these studies, which might be associated with many factors, such as differences in clinical sample sources, sample processing methods, bioinformatics analyses, detection methods, and post-analytical validation.

### Dysregulation and molecular mechanisms of circRNAs in RCC

Some circRNAs have been identified as key molecular regulators in the pathogenesis of RCC. The expression and functions of dysregulated circRNAs in RCC are listed in Table 1. These circRNAs participate in diverse processes of RCC pathogenesis, including proliferation, apoptosis, migration, invasion, epithelial-mesenchymal transition (EMT). Here, we discuss these circRNAs and their cellular functions and pathogenic mechanisms in RCC.

**Table 1:** Overview of deregulated circRNAs in renal cell carcinoma (RCC)

CircRNA	Expression Change	Function	Target microRNA	miRNA target genes/protein	Reference
**circPCNXL2**	up	proliferation (+); invasion (+)	miR-153	ZEB2	[34]
**circ-0039569**	up	Proliferation (+); migration (+); invasion (+)	miR-34a-5p	CCL22	[35]
**circ-ZNF609**	up	proliferation (+); invasion (+)	miR-138-5p	FOXP4	[36]
**circNRIP1**	up	proliferation (+); migration (+)	miR-505	AMPK and PI3K/AKT/mTOR	[37]
**circFNDC3B**	up	cell viability (+); migration (+)	miR-99a	JAK1/STAT3 and MEK/ERK	[38]
**circ-001895**	up	proliferation (+); apoptosis (−); migration (+); invasion (+)	miR-296-5p	SOX12	[39]
**circ-EGLN3**	up	proliferation (+); apoptosis (−); migration (+); invasion (+)	miR-1299	IRF7	[40]
**circ-000926**	up	proliferation (+); migration (+); invasion (+); EMT (+)	miR-411	CDH2	[41]
**circ-ZNF652**	up	proliferation (+); apoptosis (−); EMT (+)	miR-205	Ras/Raf/MEK/ERK and JAK1/STAT3	[42]
**circ-0035483**	up	autophagy (+); tumor growth (+); gemcitabine resistance (+)	miR-335	CCNB1	[43]
**circ-ABCB10**	up	proliferation (+); apoptosis (−); migration (+)	__	__	[44]
**circ-0072309**	down	proliferation (−); apoptosis (+); migration (−); invasion (−)	miR-100	PI3K/AKT and mTOR	[45]
**circ-AKT3**	down	migration (−); invasion (−); EMT (−)	miR-296-3p	E-cadherin	[46]
**cRAPGEF5**	down	proliferation (−); migration (−); invasion (−)	miR-27a-3p	TXNIP	[47]
**circ-0001451**	down	proliferation (−); apoptosis (+)	__	__	[48]
**circHIAT1**	down	migration (−); invasion (−)	miR-195-5p miR-29a-3p miR-29c-3p	CDC42	[49]
**circATP2B1**	down	migration (−); invasion (−)	miR-204-3p	FN1	[50]

**Fig. 2:**
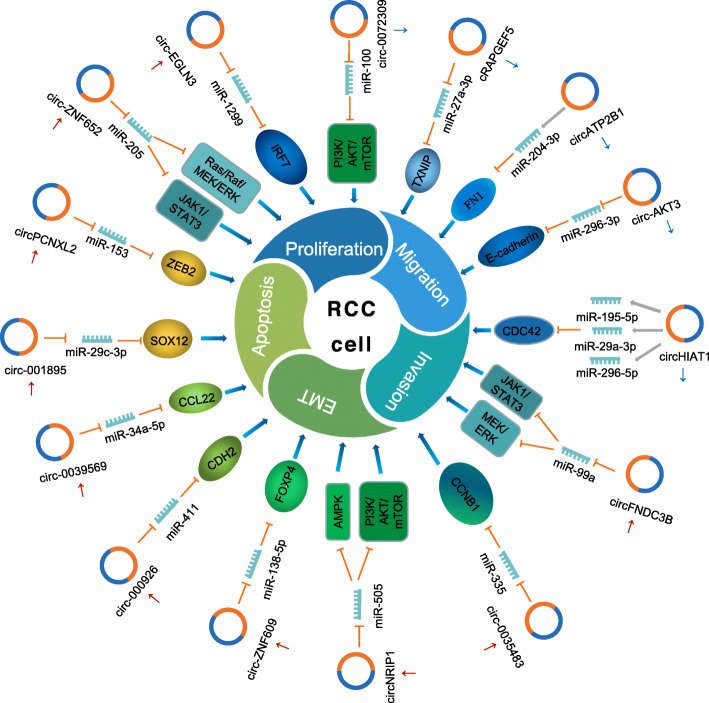
Roles and regulatory pathways of RCC-related circRNAs. The schematic diagram shows the roles of circRNAs in RCC progression and the involvement of circRNAs in the miRNA-associated gene regulatory pathways

Most circRNAs promote the development and progression of RCC through circRNA-miRNA-mRNA interaction networks **(**Fig. 2**)**. Some circRNAs act as tumor promoters in RCC cells. For instance, circ-ZNF609 was highly expressed in multiple RCC cell lines compared with the normal renal epithelial cell line KiMA [36]. Further, circ-ZNF609 markedly promoted cell proliferation and invasion in RCC cells. Mechanistically, circ-ZNF609 can work as a miR-138-5p molecular sponge, which subsequently increases levels of FOXP4, the known target of miR-138-5p. This study revealed a critical role of the circ-ZNF609/miR-138-5p/FOXP4 axis in RCC in vitro; however, its function in RCC progression in vivo awaits further investigation. Interestingly, circ-ZNF609 can be translated into a protein [85], in a process that may be regulated by m^6^A modification [86]. Further investigation is needed to show whether this mode of circ-ZNF609 regulation also contributes to RCC. EMT plays a key role in tumor invasion and metastasis in multiple cancers, including RCC. Cadherin 2 (CDH2, also known as N-cadherin) is a marker of EMT and a major contributor to RCC aggressiveness that can be directly targeted by miR-411 [87–89]. A recent study reported that a novel circRNA (circ_000926) increased CDH2 expression by inhibiting miR-411 in RCC cells [41]. Moreover, silencing circ_000926 resulted in reduced cell proliferation, migration, and invasion in vitro, and markedly suppressed the growth and metastasis of RCC tumors in vivo. These results reveal a novel circ_000926/miR-411/CDH2 regulatory axis in RCC tumorigenesis and metastasis. Other circRNAs have tumor suppressor roles in RCC cells [45–50]. Recently, the expression and function of cRAPGEF5 have implicated it as a novel tumor suppressor in RCC [47]. Reduced cRAPGEF5 expression in RCC was related to aggressive clinical characteristics. Functionally, silencing cRAPGEF5 promoted tumor growth and metastasis. RNA immunoprecipitation and in vitro biochemical assays showed that cRAPGEF5 functioned as an oncogenic miR-27a-3p sponge, which targets the tumor suppressor gene *TXNIP*. Like cRAPGEF5, circAKT3, which is transcribed from the *AKT3* gene, was significantly reduced in RCC tumors and cell lines [46]. Overexpression of circAKT3 significantly inhibited ccRCC cell migration and invasion by sponging miR-296-3p, resulting in increased E-cadherin expression. Several other regulatory cascades comprising circRNA, miRNA, and mRNA have been reported in RCC including circPCNXL2/miR-153/ZEB2 [34], circ_0039569/miR-34a-5p/CCL22 [35], hsa_circ_001895/miR-296-5p/SOX12 [39], and circEGLN/miR-1299/IRF7 [40].

Interestingly, some circRNAs can function as miRNA “reservoirs” to regulate RCC progression. The androgen receptor (AR) plays critical roles in the progression and metastasis of several hormone-regulated cancers including RCC. Wang et al. investigated the potential effects of functional interactions between AR and circRNAs in ccRCC [49]. Interestingly, circHIAT1 increased miR-195-5p/29a-3p/29c-3p activity by serving as a miRNA “reservoir”, thereby inhibiting AR-dependent migration and invasion of ccRCC cells. More recently, the novel circATP2B1 was also shown to regulate RCC progression by acting as a miRNA reservoir [50]. This study revealed that estrogen receptor β (ERβ) can promote ccRCC invasion by regulating the circATP2B1/miR-204-3p/FN1 signaling pathway. Overall, these studies illustrate that circRNAs have significant regulatory functions in RCC tumorigenesis.

In addition to miRNA regulation, recent evidence has demonstrated that some circRNAs regulate the proliferation and progression of RCC via cancer-associated signaling pathways (Table 1). For example, circNRIP1 plays an inhibitory role in RCC by regulating the adenosine monophosphate-activated protein kinase (AMPK) and PI3K/AKT/mTOR pathways [37]. Janus kinase/signal transducer and activator of transcription 3 (JAK/STAT3) and mitogen-activated protein/extracellular signal-regulated kinase (MEK/ERK) signaling pathways are involved in RCC development. Chen et al. showed that circFNDC3B promoted cell viability and migration in RCC by activating the JAK1/STAT3 and MEK/ERK pathways [38]. Similarly, circZNF652 increased proliferation and EMT of RCC cells by regulating the Ras/Raf/MEK/ERK and JAK1/STAT3 signaling pathways [42]. Although few circRNAs have been reported to play a role in RCC-associated signaling pathways to date, this list will undoubtedly continue to grow as more studies on the pathogenesis of RCC are carried out.

### circRNAs as diagnostic and prognostic biomarkers for RCC

Screening and early diagnosis of RCC are critical for improving treatment efficacy and reducing the mortality of patients with RCC and have been identified as a research priority [90]. RCC can remain undetected for an extended period and only a small number of patients are diagnosed with RCC after presenting with classical symptoms, such as hematuria, flank pain, and a palpable abdominal mass [91]. Recently, with the widespread use of computed tomography (CT) and magnetic resonance imaging (MRI), incidental renal masses are increasingly being detected. However, there is an urgent need for specific biomarkers to allow for the early identification of postoperative RCC recurrence and metastasis. Notably, serum and urine analyses can be promising diagnostics because they are noninvasive and inexpensive.

circRNAs have great potential as a novel, attractive class of ncRNA biomarkers in liquid biopsy because they are resistant to RNase R digestion and remain stable in the circulation. circRNAs are abundant in body fluids, such as saliva, blood, and urine [92–95], and they can be detected using relatively inexpensive quantitative reverse transcriptase-polymerase chain reaction (qRT-PCR) assays [96]. Recent studies using clinical RCC samples demonstrated the abnormal expression, higher disease specificity, and clinical relevance of specific circRNAs, making them ideal candidates for RCC diagnosis. For instance, hsa_circ_0001451 was significantly decreased in ccRCC samples, and its level was linked to clinicopathological features and overall survival (OS), indicating the potential of this circRNA as a diagnostic marker for ccRCC. hsa_circ_0001451 had an area under the receiver operating characteristic curve (AUC-ROC) of 0.704 for discriminating ccRCC from normal controls, with sensitivity and specificity of 0.755 and 0.608, respectively [48]. Franz et al. identified dysregulated circRNAs in ccRCC tissues by using a whole-genome microarray [84]. Three circRNAs (circEGLN3, circNOX4, and circRHOBTB3) were identified and clinically validated by qRT-PCR. Moreover, the AUC-ROC of circNOX4 and circRHOBTB3 in RCC tissues were 0.81 and 0.82, suggesting that both could be used as potential diagnostic biomarkers. Importantly, circEGLN3 had AUC-ROC of 0.98, indicating remarkably reliable diagnostic value. Further, the combined detection of circEGLN3 and linEGLN3 increased the AUC-ROC to 0.99, with 95% sensitivity and 99% specificity. These results indicated that the diagnostic value of a combination of circRNAs is higher than that of individual circRNAs.

Additionally, some circRNAs could be used as prognostic biomarkers. Huang et al. showed that circ-ABCB10 promoted RCC cell growth and suppressed apoptosis in vitro [44]. Moreover, circ-ABCB10 was markedly increased in RCC tissues and it was associated with pathologic grade and tumor-node-metastasis (TNM) stage. Therefore, circ-ABCB10 could be a prognostic factor for RCC. Another example is cRAPGEF5 [47]. In a study including 245 RCC cases, cRAPGEF5 downregulation was significantly associated with large tumor size, advanced TNM stage, and distant metastasis. Moreover, the level of cRAPGEF5 was correlated with aggressive tumor characteristics and poor OS and relapse-free survival (RFS) in patients, indicating that cRAPGEF5 could be used as a prognostic biomarker for RCC. circPRRC2A expression was increased and it was positively correlated with advanced TNM stage and lymph node metastasis in patients with RCC, indicating an oncogenic role. Furthermore, multivariate analyses indicated that circPRRC2A expression level was an independent risk factor for OS, together with RCC tumor size, pT stage, and Fuhrman grade. Kaplan-Meier survival curves showed that high circPRRC2A expression was associated with degreased with metastasis-free survival. These analyses also indicated that circPRRC2A may be a good prognostic biomarker for RCC [97].

The above examples demonstrated that some circRNAs could be promising biomarkers for the diagnosis and prognosis of RCC. However, these circRNAs are differentially expressed in tissues but are undetectable in plasma or serum. Can we develop liquid biopsy circRNAs biomarkers to distinguish healthy individuals from RCC patients and to correlate circRNA levels with tumor stages and metastasis risk? It is worth noting that there are still challenges hindering the clinical application of circRNAs as biomarkers. Monitoring biomarker research requires complex, large-scale prospective studies including the collection of continuous samples and the definition of time points and intervals, before circRNAs can be recommended for clinical use. Additionally, the normalization of circRNAs is another central point in liquid biopsies, which requires repeated tests to choose the optimal time and the cut-off value for their application to be compatible with the needs of patients. Despite these challenges, circRNAs are highly promising biomarker candidates, which can open more possibilities in RCC diagnosis and prognosis.

### circRNAs as potential therapeutic targets for RCC

The emerging importance of circRNAs in the initiation and progression of RCC makes them an attractive therapeutic option [98–100]. For example, circHIAT1 and circATP2B1 could function as metastasis inhibitors to prevent AR- or ERβ-induced ccRCC cell migration and invasion. Targeting these newly identified AR-circHIAT1- miR-195-5p/29a-3p/29c-3p/CDC42 and ERβ-circATP2B1-mediated miR-204-3p/FN1 signaling cascades can be a potential new route for development of therapies for metastatic ccRCC [49, 50].

Resistance to chemotherapeutic drugs is an intractable problem in RCC treatment. Gemcitabine is a novel type of drug that can selectively target tyrosine kinases [101]. Although gemcitabine has greatly improved RCC treatment, drug resistance consistently emerges after long-term application. Elucidating the molecular mechanism of gemcitabine resistance is important for RCC treatment. Recently, Yan et al. found that hsa-circ_0035483 was aberrantly expressed in RCC and enhanced gemcitabine resistance by regulating the hsa-miR-335/CCNB1 signaling pathway, thereby impairing chemotherapy effectiveness [43]. These results suggested that hsa_circ_0035483 could be a promising target for preventing gemcitabine resistance in RCC therapy. However, studies on circRNAs in RCC resistance to chemotherapy are still at a nascent stage. The connection between specific circRNAs and resistance to other classic drugs used in RCC treatment is still not clear. Undoubtedly, substantial efforts will be undertaken to reveal the function of circRNAs in RCC chemoresistance. Comprehensive evaluation of circRNA function in RCC will advance our knowledge of the mechanisms underlying RCC chemoresistance.

Several strategies for RCC treatment, which take into account the important functional roles of circRNAs as miRNA sponges, have been proposed. First, inhibiting or restoring circRNA function to regulate miRNAs and subsequently, their downstream targets, may be useful in RCC therapy. Thus far, several methods of inhibiting circRNA function have been developed [102], including siRNA, antisense oligonucleotide (ASO), CRISPR/Cas9-mediated knockout, and disrupting the association of circRNAs with miRNAs or RBPs by saturating the conserved binding sites on circRNAs. In contrast, when circRNAs function as sponges for RBPs or miRNAs, restoring circRNA expression may inhibit abnormal proliferation or induce apoptosis. Thus, artificial circRNA sponges may be an effective approach to restore circRNA functions, providing a new strategy for drug development based on targeting miRNAs [76, 103, 104]. Furthermore, endogenous circRNAs are stable and have tissue- and cell-specific expression, suggesting that targeting circRNAs may be a future direction for gene therapy with potentially lower toxicity than that of synthetic molecules, such as siRNAs and synthetic drugs.

Exploring the unique properties of circRNAs is a novel frontier in RCC treatment. Engineered circRNAs could be an accurate and effective method for delivering therapeutic agents. For instance, high-quality protein translation was achieved by using an artificial circRNA and protein half-life was extended threefold [105]. In addition, the Tornado expression system has been developed to efficiently increase circRNA expression in cells [106]. At present, although such circRNAs have not been found in RCC, further research may identify circRNA with promising application potential for RCC therapy. Overall, research on circRNAs as potential therapeutic targets or useful tools is a rapidly developing field in oncology.

### Challenges and future perspectives

Currently, interest in circRNAs is growing and their role in RCC is becoming better understood. However, the clinical application of circRNAs in RCC remains largely unexplored and further research is needed prior to incorporating circRNAs into clinical practice.

Only a few circRNAs have defined biological functions and molecular mechanisms in RCC and the exact mechanisms of circRNA circularization, degradation, and cellular localization in RCC need further investigation. Due to potential alternative splicing, the internal structure of circRNAs has remained ill-defined, which has impeded research on circRNA function. Notably, Feng et al. developed a new algorithm, CircSplice, which could identify internal alternative splicing in circRNAs and compare differential circRNA splicing events between different conditions [107]. The application of this algorithm in ccRCC characterized patterns of cancer-specific circRNA alternative splicing (circ-AS), providing a useful resource to explore the functional and regulatory roles of circRNAs in RCC. At present, most studies on circRNAs are focused on their function as miRNA sponges that regulate the expression of the targeted genes. In addition to this regulatory mechanism, other mechanisms may also be involved in the regulation of circRNA function. It is unclear whether circRNAs can be regulated through different molecular regulatory mechanisms at the same time. Further, many studies have confirmed that the tumor microenvironment can impact tumor initiation and progression; however, the effects of circRNAs on the RCC microenvironment have remained elusive until recently [108, 109]. Therefore, elucidating the role of circRNAs in the tumor microenvironment will further clarify the underlying mechanisms of RCC initiation and progression, thereby identifying possible targets for therapeutic intervention.

Although several circRNAs can be potential tumor biomarkers, current research investigating circRNAs as RCC biomarkers is limited. Currently, most circRNA biomarkers may not be suitable for clinical application since they are lacking in sensitivity or specificity. Importantly, more clinical studies on circRNAs as biomarkers should be carried out and will require standardized techniques and bioinformatics methods to reliably detect circRNAs.

Ultimately, more research is needed on how to efficiently deliver circRNAs to recipient cells to regulate cancer progression without immunologic rejection and with sustained long-term effects. Exosomes may greatly expand the applications of circRNAs. Exosomes are small membrane vesicles released by most cell types including tumor cells, which can act as natural intercellular communication mediators by transporting signaling molecules, including circRNAs, to target tissues. Recently, exosomes have been confirmed to contain stable and abundant circRNAs [110]. Intriguingly, serum exosomal circRNAs can distinguish patients with colon cancer from healthy controls [110]. However, the biological functions of exosomal circRNAs in RCC progression need further exploration.

## Conclusions

In this review, we discussed and summarized the recent discoveries and research progress on RCC-associated circRNAs, and further emphasized their potential clinical applications, which provides a new direction for developing RCC diagnostics and therapies. However, c circRNAs contribute to RCC initiation, and progression is not fully understood. It is highly anticipated that circRNA-based diagnostic and therapeutic interventions for RCC will emerge in the near future.

## Data Availability

Not applicable.

## Abbreviations

RCC: Renal cell carcinoma
circRNAs: Circular RNAs
ncRNAs: Non-coding RNAs
miRNAs: MicroRNAs
lncRNAs: Long-noncoding RNAs
tsRNAs: Transfer RNA [tRNA]-derived small RNAs
piRNAs: PIWI-interacting RNAs
ecircRNAs: Exonic circRNAs
ElciRNAs: Exon-intron circRNAs
ciRNAs: Intronic circRNAs
tricRNAs: CircRNAs formed by the splicing of pre-tRNA
Pol II: RNA polymerase II
snRNP: Small nuclear ribonucleoprotein
mecciRNA: Mitochondrial circRNA
NASH: Nonalcoholic steatohepatitis
mito-NP: Mitochondria-targeting nanoparticles
RBPs: RNA-binding proteins
ceRNA: Competing endogenous RNA
ccRCC: Renal clear cell carcinoma
EMT: Epithelial-mesenchymal transition
TNM: Tumor-node-metastasis
AR: Androgen receptor
ERβ: Estrogen receptor beta
qRT-PCR: Quantitative reverse transcriptase-polymerase chain reaction
OS: Overall survival
RFS: Relapse-free survival
ROC: Receiver operating characteristic
GEO: Gene Expression Omnibus
ASOs: Anti-sense oligonucleotides
CRISPR/Cas9: Clustered regularly interspaced short palindromic repeats-associated nuclease Cas9
circ-AS: CircRNA alternative splicing
